# Polymorphic Membrane Protein 17G of *Chlamydia psittaci* Mediated the Binding and Invasion of Bacteria to Host Cells by Interacting and Activating EGFR of the Host

**DOI:** 10.3389/fimmu.2021.818487

**Published:** 2022-01-31

**Authors:** Xiaohui Li, Zonghui Zuo, Yihui Wang, Johannes H. Hegemann, Cheng He

**Affiliations:** ^1^ Key Lab of Animal Epidemiology and Zoonoses of Ministry of Agriculture and Rural Affairs, College of Veterinary Medicine, China Agricultural University, Beijing, China; ^2^ Tianjin Key Laboratory of Agricultural Animal Breeding and Healthy Husbandry, College of Animal Science and Veterinary Medicine, Tianjin Agricultural University, Tianjin, China; ^3^ Department of Biology, Institute for Functional Microbial Genomics, Heinrich-Heine-Universität Düsseldorf, Düsseldorf, Germany

**Keywords:** *C. psittaci*, Pmp17G, EGFR, adhesion, invasion

## Abstract

*Chlamydia psittaci* (*C. psittaci*) is an obligate intracellular, gram-negative bacterium, and mainly causes systemic disease in psittacine birds, domestic poultry, and wild fowl. The pathogen is threating to human beings due to closely contacted to employees in poultry industry. The polymorphic membrane proteins (Pmps) enriched in *C. psittaci* includes six subtypes (A, B/C, D, E/F, G/I and H). Compared to that of the 1 *pmpG* gene in *Chlamydia trachomatis* (*C. trachomatis*), the diverse *pmp*G gene-coding proteins of *C. psittaci* remain elusive. In the present study, polymorphic membrane protein 17G (Pmp17G) of *C. psittaci* mediated adhesion to different host cells. More importantly, expression of Pmp17G in *C. trachomatis* upregulated infections to host cells. Afterwards, crosstalk between Pmp17G and EGFR was screened and identified by MALDI-MS and Co-IP. Subsequently, EGFR overexpression in CHO-K1 cells and EGFR knockout in HeLa 229 cells were assessed to determine whether Pmp17G directly correlated with EGFR during Chlamydial adhesion. Finally, the EGFR phosphorylation was recognized by Grb2, triggering chlamydial invasion. Based on above evidence, Pmp17G possesses adhesive property that serves as an adhesin and activate intracellular bacterial internalization by recognizing EGFR during *C. psittaci* infection

## Introduction

*Chlamydia psittaci* (*C. psittaci*) is an important zoonotic agent with a wide host spectrum (1). It causes systemic infection, especially in birds, which leads to profound economic losses in poultry annually (2). For reporting cases of *C. psittaci* infection, a national surveillance program in the United States (U.S.) has been operational since 1998. However, no surveillance or case reporting system has been implemented for identifying human chlamydiosis in China. In recent years, case of human psittacosis has increased gradually, and 15 workers were reported to be infected in a U.S. poultry slaughterhouse in 2018, indicating a high risk of zoonotic transmission of Psittacosis (3). In recent years, human psittacosis has increased gradually in China due to implementation of next generation genomic sequencing. Infected hosts (both animal and human) show a diversity of clinical signs, from asymptomatic disease for a large portion of infected organisms to multiple organ failure, sepsis, and death (4). However, the mechanism by which the target hosts is infected and tissue tropism exhibited by *C. psittaci* upon infection remain unknown.

Attachment and invasion to host cells are milestones in *Chlamydia* infection and growth. *Chlamydia* has evolved capabilities to invade host cells *via* receptors and multiple pathways, similar to *Legionella pneumophila* (5), *Brucella* (6) and other intracellular pathogens (7). Multiple outer membrane molecules on *Chlamydia* have been identified and shown to be associated with adhesion, such as major outer membrane protein (MOMP) (8, 9), lipopolysaccharide (LPS) (10), CT017(Ctad1) (11), and outer membrane complex protein B (OmcB) (12–14). More importantly, polymorphic membrane proteins (Pmps) are dominant adhesins that elicit chlamydial infection (15–17). In addition, Pmps are immunodominant and located on the elementary bodies (EBs) surface of *Chlamydia trachomatis* (*C. trachomatis*) and *Chlamydia pneumoniae* (*C. pneumoniae*) (18–21). However, the role of Pmps in *C. psittaci* has been rarely studied, and Pmps interaction mechanism with host cells remains unclear. Regarding diverse *pmpG* genes and transcription across species, only one *pmpG* gene has been identified in *C. trachomatis* genome, and the *pmpG* gene encoding amino acid sequences in different *C. trachomatis* serovars is highly conserved. For instance, the identity of PmpG in serovar E and L2 is 98%. However, 37%, 28%, and 33% similarity has been found among Pmp7G, Pmp17G and Pmp21G in *C. psittaci*. The virulence and pathogenicity are similar to those of *C. psittaci* 6BC and more homologous *pmpG* genes have evolved, as indicated by the numbers of functional sequences. Interestingly, the highly homologous *pmpG* genes of the *C. psittaci* Cal10 strain are expressed during the late life cycle, while low levels of homologous *pmpG* genes, identified by a relatively low level of transcripts, are found in the whole cycle (22). These results indicate that *pmpG* genes play different roles during the life cycle and that highly homologous PmpG proteins may be involved in the assembly of EBs and critical for bacterial adhesion to host cells. According to a recent report, Pmp17G is associated with host adaptations as an adhesin (16). However, the mechanism of Pmp17G-mediated attachment of bacteria to host cells remains unclear. Therefore, we hypothesize that Pmp17G in *C. psittaci* 6BC is recognized and interacts with potential receptor(s), thereby mediating tissue tropism, multi-host infection and diverse pathogenicity. PmpG-mediated adhesion to and invasion into host cells by recognizing specific receptors is urgently needed during *C. psittaci* infection.

## Materials and Methods

### Cell Lines and Bacterial Strains

HeLa 229 cells (No. TCHu 20) and Vero cells (No. GNO10) were purchased from the National Collection of Authenticated Cell Cultures (NCACC; Beijing, China). HEp-2 cells (ATCC, No. CCL-23) and DF-1 cells (ATCC, No. CRL-12203) were maintained in 8% foetal bovine serum (FBS; Gibco, Beijing, China)/Dulbecco’s modified Eagle’s medium (DMEM) (Solarbio Life Science Ltd., Beijing, China), and Chinese hamster ovary (CHO-K1; No. SCSP-507) cells were purchased from NCACC and cultured in Ham’s F-12K (Invitrogen, Beijing, China) supplemented with 10% FBS. All cell lines were cultured at 37°C with a 5% CO_2_ flow. *C. psittaci* 6BC (GenBank: CP002549.1) was kindly donated by Professor Yimo Wu (University of South China, Hunan, China) and cultivated, purified and titrated as previously described (23). *C. trachomatis* L2 (Institute for Functional Microbial Genomics, Heinrich-Heine-Universität, Düsseldorf, Germany) were propagated in HEp-2 cells as described (15).

### Antibodies and Reagents

The following antibodies were used in this study: anti-GAPDH (MA5-15738), anti-6×His (MA1-21315), anti-HA (26183) and anti-flag (MA1-91878) mouse monoclonal antibodies (mAbs) and goat anti-mouse IgG H+L (HRP) secondary antibody (31430), which were commercially obtained from Thermo Fisher Scientific (Beijing, China), and anti-EGFR (sc-373746), and anti-phospho-EGFR (1068) (sc-81488) and anti-Grb2 (sc-8034) mAbs, which were purchased from Santa Cruz Biotechnology (Santa Cruz, Shanghai, China). The remaining anti-EGFR rabbit mAbs (4267; CST, Beijing, China) and goat anti-rabbit IgG H&L (HRP) secondary antibody (ab6721; Abcam, Beijing, China) were also commercially available products. Anti-GroEL rabbit pAbs, anti-MOMP mouse mAbs and anti-S1 rabbit pAbs were from Heinrich-Heine-Universität and described previously (11).Anti-MOMP mouse mAbs were prepared by immunizing mice with recombinant MOMP from *C. psittaci* 6BC in our laboratory as described previously (19). Anti-Pmp17G rabbit polyclonal antibodies (pAbs) were prepared in rabbits inoculated with recombinant Pmp17G protein that was expressed and purified from *E. coli* BL21 cells (TransGen Biotech Ltd., Beijing, China). The antibodies were purified using HiTrap™ rProtein AF (GE, Beijing, China). The specificities of the MOMP monoclonal antibodies and polyclonal antibodies against Pmp17G were identified with ELISAs as described previously (19).

### Plasmid Construction and Protein Preparation

The *pmp17G* gene was amplified from EBs of C. *psittaci* 6BC and then subcloned into a pKM255 shuttle vector(Heinrich-Heine-Universität, Düsseldorf, Germany), a pET28a prokaryotic vector or a pCMV-C-HA eukaryotic vector (Beyotime Biotech Ltd., Shanghai, China), respectively. Targeted ectodomain of Pmp17G protein was listed in **Figure S2**. cDNA samples of EGFR from HeLa 229 cells were amplified by RT-PCR and then subcloned into a pCMV-C-flag vector (Beyotime Biotech Ltd, Shanghai, China), named pCMV-EGFR-N-flag. Four restriction enzymes and T4 DNA ligase were obtained from a commercial company (New England Biolabs, Beijing, China). PCR was performed using Q5 High-Fidelity DNA polymerase (New England Biolabs, Beijing, China). PCR primers were listed in **Table S1**. Both recombinant Pmp17G protein and MOMP protein were induced by isopropyl-β-D-thiogalactopyranoside (IPTG; Beyotime Biotech Ltd., Shanghai, China) in 500 ml of cultured *E. coli* BL21 cells. After purification with Ni-NTA Resin(88221, Thermo Fisher Scientific, Beijing, China), the recombinant MOMP protein and Pmp17G were confirmed by SDS-PAGE and Western blots as described previously (19).

### Adhesion Assays With the Pmp17G Protein

First, HeLa 229, Vero and DF-1 cells were grown in 24-well plates for 24 h. Then, the culture media were removed, and the cells were washed 3 times with cold PBS and then incubated with 200 µl of Pmp17G (200 µg/ml) to cover the surface of the cells. Subsequently, the cultures were incubated at 37°C with 5% CO_2_ flow and monitored at 15, 30, 60, 90, and 120 min. Finally, the cell cultures were washed 5 times with PBS, and then, the cells were lysed in 50 µl of RIPA buffer with phenylmethylsulfonyl fluoride (PMSF; Beyotime Biotech Ltd, Shanghai, China). The lysed solutions were centrifuged at 12,000 g for 10 min, and then, the supernatants were boiled with loading buffer. Finally, 10 µl of the cell lysate was identified by SDS-PAGE and Western blots.

### Expression of *C. psittaci*-Specific Pmp17G in *C. trachomatis* L2 (*C. trachomatis* L2 (pKM255::*pmp17G)*

Chlamydial transformation was performed as described previously (24). Briefly, the pKM255::*pmp17G* plasmid (Institute for Functional Microbial Genomics, Heinrich-Heine-Universität, Düsseldorf, Germany), was extracted from a Dam- and Dcm-methylase-deficient strain of *E. coli* (GM-48), using a plasmid mega kit (Qiagen, Hilden, Germany). Penicillin (1µg/µl) was used for the selection of transformed *C. trachomatis* L2. A mixture of *C. trachomatis* L2 EBs and pKM255::*Pmp17G* was incubated in calcium chloride buffer (10 mM Tris, 50 mM calcium chloride, pH 7.4) for 30 min at room temperature. Subsequently, *C. trachomatis* L2 was mixed with pKM255::*pmp17G* and incubated with HEp-2 cells in calcium chloride buffer for 1 h at room temperature. The total calcium chloride mixture was discarded and replaced with fresh 10% FBS/DMEM containing 2 µg/ml cycloheximide. After 24 hpi, penicillin (1 µg/µl) was added and further incubated for 36 h at 37°C under 5% CO_2_. Afterwards, the infected epithelial cells were scraped and lysed with glass beads for next passages untill inclusions were observed obviously. Positive expression of Pmp17G in *C. trachomatis* L2 was identified to bind anti-flag antibody by immunofluorescence (IF). Furthermore, location of the Pmp17G was analyzed using different detergent extractions as described previously (11).

### Co-IP of Overexpressed Proteins and Surface Complexes

For overexpressing proteins, HeLa 229 cells were transfected with plasmids containing the *EGFR* gene and/or *pmp17G* gene using Lipofectamine™ 3000 (Invitrogen, Beijing, China) according to the manufacturer’s protocol. As for immunoprecipitation(IP) of surface complexes, HeLa 229 cells were grown to 90% confluency, and the cell cultures were washed with PBS and incubated with 200 µg/ml purified Pmp17G in FBS-free DMEM at 37°C for 2 h. Twenty-four hours after transfection or 2 h after incubation, cell cultures were rinsed 3 times with PBS and then lysed in IP lysis buffer (containing 1% NP-40, 1% Triton X-100, 20 mM Tris-HCl at pH 7.5, 150 mM NaCl, 1 mM Na_3_VO_4_, 5 mM NaF, and 2 mM EDTA) and protease inhibitor cocktail (Beyotime Biotech Ltd, Shanghai, China) at 4°C for 30 min. Then, cell lysates were precleared with standard IgG (rabbit sera) (Solarbio Life Science, Beijing, China) together with protein A/G PLUS-agarose (Santa Cruz, Shanghai, China) at 4°C for 30 min. Then, the precleared cell lysates were used for IP reactions. Co-IP reactions were carried out by mixing anti-EGFR mAbs or anti-Pmp17G pAbs at 4°C for 6 h and then washing the lysates 3 times with IP buffer. Finally, the target proteins were identified by Western blots using HA-tagged or flag-tagged antibodies.

### Blocking Infection With Pmp17G or Anti-Pmp17G Antibody

HeLa 229 cells were incubated with Pmp17G (200 µg/ml) at 37°C for 2 h. Afterwards, the unbound proteins were discarded after washing 5 times with PBS, and the cells were infected with *C. psittaci* 6BC at an MOI of 5 in medium containing 8% FBS/DMEM and 2 µg/ml cycloheximide (Sigma-Aldrich, Shanghai, China) and then incubated at 37°C for 36-48 h. To confirm blocking infection, *C. psittaci* 6BC EBs were diluted with SPG solution (containing 0.25 M sucrose, 10 mM sodium phosphate, and 5 mM L-glutamic acid, pH 7.2-7.5) to a final concentration of 1.0×10^6^ inclusion-forming units (IFUs)/ml. The diluted EBs were incubated with purified anti-Pmp17G pAbs at 50 µg/ml, and the cultured EBs were allowed to react at room temperature for 2 h using a shaking machine. Later, HeLa 229 cells were infected with the above cultures at 37°C for 36-48 h. IFUs were quantified using a monoclonal antibody against MOMP and IF microscopy. The adhesion studies and internalization assays were carried out using Pmp17G-coated fluorescent beads as described previously (25).

### Effect of EGFR Regulation on *C. psittaci* Infection

To identify the effect of EGFR on *C. psittaci* 6BC infection, HeLa 229 cells were pretreated with different concentrations of cetuximab (Sigma-Aldrich, Shanghai, China), an anti-EGFR antibody, at 37°C for 2 h. Then, the unbound antibodies were removed from the reaction followed by washing 5 times with PBS, and finally, cell cultures were infected with *C. psittaci* 6BC at an MOI of 5 and incubated at 37°C for 36-48 h. In another experiment, EGFR in HeLa 229 cells was knocked down *via* transfection with EGFR siRNA (Santa Cruz, Shanghai, China) according to the manufacturer’s protocol, and the knockdown efficiency was confirmed by Western blots. Then, the transfected HeLa 229 cells were exposed to *C. psittaci* 6BC at an MOI of 5 at 37°C for 36-48 h. To construct overexpressed EGFR in CHO-K1 cells, the cells were transfected with pCMV-EGFR-N-flag at 37°C for 24 h. Subsequently, the overexpressed cells were infected at an MOI of 5 at 37°C for 36-48 h. IFUs were determined as described above.

### Effect of Pmp17G Recognition of EGFR on the Phosphorylation of Try1068 and Grb2

HeLa 229 cells were cultivated with 8% FBS at 37°C for 24 h and then replaced with FBS-free medium for 12 h. Subsequently, cell cultures were stimulated with different concentrations of Pmp17G (from 25 to 500 µg/ml) or *C. psittaci* 6BC (MOI from 0.1 to 100) at 37°C for 2 h. Then, the cell cultures were washed 5 times with cold PBS, and the cells were lysed in 50 µl of IP lysis buffer with protease and phosphatase inhibitor cocktail. The lysed solutions were centrifuged at 12,000 g for 10 min, and then, the supernatants were boiled with 1x loading buffer for 10 min. Later, 10 µl of the cell lysates were identified by SDS-PAGE and Western blots. The supernatants were tested for EGFR, pEGFR_1068_, and Grb2 expression using anti-EGFR, anti-pEGFR_1068_, anti-Grb2 and anti-GAPDH antibodies, respectively. The optimal concentrations of Pmp17G and EBs of *C. psittaci* 6BC were applied in a time-dependent manner. HeLa cells were infected with 200 µg/ml Pmp17G or *C. psittaci* 6BC at an MOI of 5 for different durations (from 0, 5, 10, 15, 30 to 60 min). Then, the cell cultures were treated as described above. To carry out Co-IP with EGFR at Try1068 and Grb2, HeLa cells were infected with 200 µg/ml Pmp17G or *C. psittaci* 6BC at an MOI of 5 for 30 min. The cell cultures were treated IP lysis buffer containing a protease and phosphatase inhibitor cocktail. Finally, the supernatants were subjected to IP with anti-EGFR antibodies as explained above.

### Statistical Analysis

Statistical analyses were carried out by analysis of variance (ANOVA). Multiple comparisons and differences were analyzed using the least-significant difference (LSD) method. A p-value (two-tailed) of 0.05 or less was considered to indicate significance. Analyses were carried out using GraphPad Prism software (GraphPad Software Inc., CA, USA) and Image J (National Institutes of Health, Maryland, USA).

## Results

### Pmp17G Triggered Adhesion to Multi-Host Cells, and Recombinant Pmp17G Blocked *C. psittaci* Infection

**Figure 1:**
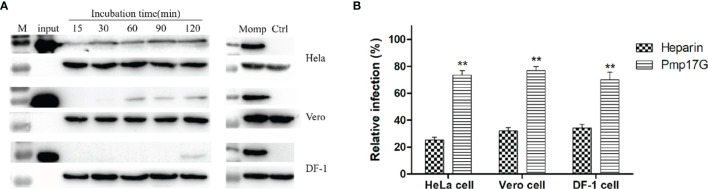
Pmp17G mediated adhesion to host cells, and recombinant Pmp17G blocked *C*. *psittaci* infection. **(A)** HeLa 229 cells, Vero cells and DF-1 cells were incubated with 200 μg/ml Pmp17G, while inactivated EBs at an MOI of 5 were used as a positive control for different durations. Positive bands were reacted with anti-His mAbs or anti-MOMP antibody, and GAPDH was the loading control. **(B)** HeLa 229 cells, Vero cells and DF-1 cells were pretreated with 200 μg/ml of Pmp17G and 500 μg/ml heparin, as the positive control for 2 h, and then exposed to *C*. *psittaci* 6BC at an MOI of 5 for 36-48 h Inclusion-forming units (IFUs) were quantified by indirect immunofluorescence (IIF). Relative Infection (%)=(IFUs of the treated group/IFUs of the PBS group)×100. Statistical analysis was performed by one-way ANOVA, and the data from 3 independent experiments were expressed as the means ± standard deviations (SD). Asterisks indicate statistical significance: **p < 0.01.

In the present study, strong Pmp17G adhesion was found to HeLa 229 cells, and secondary higher attaching capability was observed in Vero cells than DF-1 cells did. Regarding the time course, early adhesion was observed at 15 min, and an increasing attachment was correlated with prolonged incubation and the strongest signal was detected at 120 min, while secondary adhesion occurred at 60 min in Vero cells and 120 min in DF-1 cells, respectively (**Figure 1A**). Subsequently, 3 different host cells were incubated with Pmp17G, and the relative *C. psittaci* infection of HeLa 229 cells, DF-1 cells and Vero cells was reduced to 72.15%, 70.04% and 76.97%, respectively, which was higher than that of the heparin-positive group. However, no difference was found among the 3 host cells prior to treatment with recombinant Pmp17G (**Figure 1B**), indicating that Pmp17G might attach to multiple host cells and involve in tissue tropism, contributing to the wide spectrum of *C. psittaci* infection in comparison with *C. trachomatis* (15) and *C. pneumoniae* (17).

### Ectopic Expression of *C. psittaci*-Specific Pmp17G Enhanced *C. trachomatis* Infection

**Figure 2:**
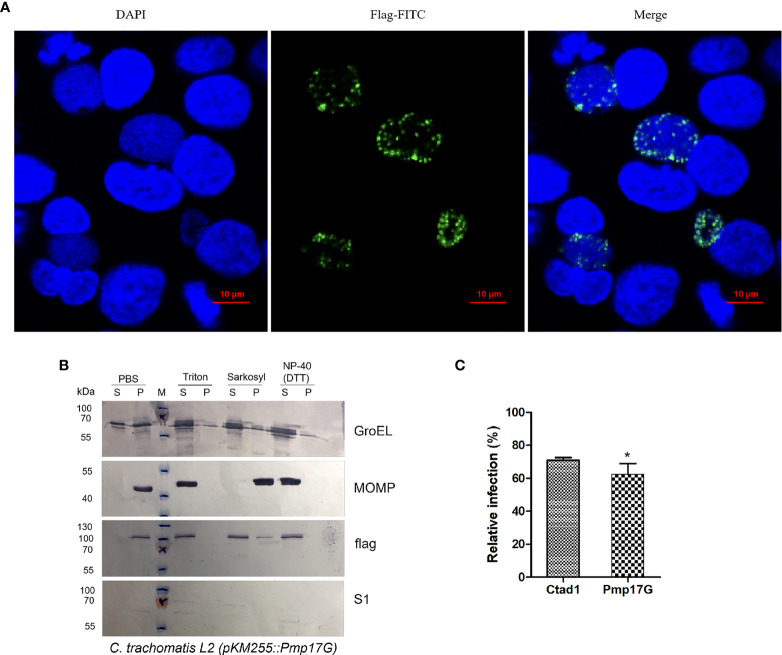
Expression of *C.psittaci*-specific PmpG17 upregulated *C*. *trachomatis* infection. **(A)** Identification of *C. psittaci*-specific PmpG17 in *C. trachomatis* L2 strain by indirect immunofluorescence at 24 hpi. **(B)** Expressions of *C psittaci*-specific PmpG17 were located both on outer membrane and cytoplasm of elementary bodies by Western blots. GroEL, a heat-shock protein located both on outer membrane surface and cytoplasm, MOMP, an outer membrane protein, and S1 protein, an intracellular protein of *C. trachomatis* L2 as the control group. Elementary bodies of *C. trachomatis* L2 (pMK255::*pmp17G*) were treated with PBS, 1% Triton-X 100, 2% Sarkosyl or N-40 with DTT, respectively. Pellet (P) and supernatant (S) fractions were prepared by centrifugation and incubated with anti-S1, anti-MOMP, anti-GroEL1 and anti-flag antibodies, respectively. **(C)** HEp-2 cells were preincubated with 200 μg/ml of recombinant Pmp17G or Ctad1 as a positive control. Afterwards, above cell cultures were inoculated with *C. trachomatis* L2 (pMK255::*pmp17G*) at an MOI of 5 for 24 hours. IFUs were quantified as above described. Relative infection (%)=(IFUs of the treated group/IFUs of the PBS group)×100. Statistical analysis was performed by one-way ANOVA, and the data from 3 independent experiments were expressed as the means ± SD. Asterisks indicate statistical significance: *p < 0.05.

Regarding the only one *pmpG* gene of *C. trachomatis*, the role of ectopic expression of *C. psittaci*-specific Pmp17G in *C. trachomatis* will be a fundamental model to elucidate the functions of the duplication of *pmpGs* in *C. psittaci*. Interestingly, expression of *C. psittaci*-specific Pmp17G in *C. trachomatis* L2 was observed using indirect immunofluorescence (IF) at 24-hour post infection (hpi) **(** **Figure 2A** **)**. Moreover, locations of *C. psittaci*-specific PmpG17 were found both on outer membrane and cytoplasm of EBs by Western blots. Meanwhile, GroEL was located both on outer membrane surface and cytoplasm, MOMP as outer membrane protein and S1 protein located in cytoplasm of *C. trachomatis* L2 were used as the control groups **(** **Figure 2B** **)**. To warrant above observation, Hep-2 cells were treated with Pmp17G or Ctad1 and infected with ectopic expression of *C. trachomatis* L2 (pKM255::*pmp17G*).Interestingly, Pmp17G displayed strong blocking infection compared to Ctad1 post infection of *C. trachomatis* L2 (pKM255::*pmp17G*) (p<0.05) **(** **Figure 2C** **)**.

### Pmp17G Directly Binded to and Interacted With the Cellular Transmembrane Protein

**Figure 3:**
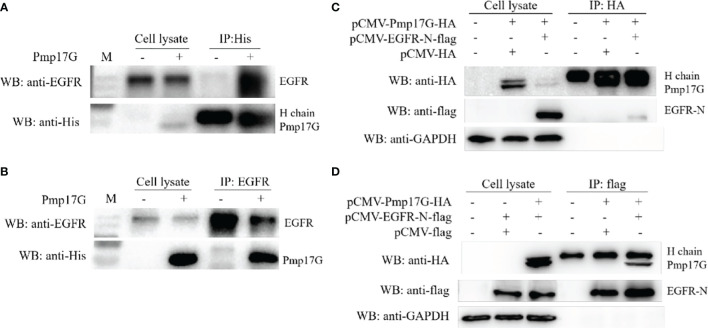
Crosstalk of Pmp17G with EGFR. **(A)** Pmp17G was incubated with HeLa 229 cells for 2 h, immunoprecipitated with anti-His mAbs, and then reacted with anti-EGFR mAbs and anti-His mAbs. **(B)** After incubation with HeLa 229 cells, IP was assayed with anti-EGFR mAbs, and then, Pmp17G-recognized EGFR was identified with anti-His mAbs and anti-EGFR mAbs using Western blots. **(C)** Co-expression of Pmp17G and EGFR in HeLa 229 cells. HeLa 229 cells were transfected with pCMV-Pmp17G-HA and pCMV-EGFR-N-flag plasmids, reacted with anti-HA antibody for IP, and then incubated with anti-flag and anti-EGFR mAbs by Western blots. **(D)** Co-expression of Pmp17G and EGFR in HeLa 229 cells was evaluated by Co-IP. HeLa 229 cells were transfected with pCMV-Pmp17G-HA and pCMV-EGFR-N-flag plasmids, immunoprecipitated with anti-flag antibody, and then identified with anti-flag and anti-EGFR mAbs using Western blots.

Pmp17G was incubated with HeLa 229 cells for 2 h, and the attached fractions in the pull-down elution using Ni-NTA were identified and analyzed by MALDI-MS **(** **Figure S1** **).** Although no difference was found in the SDS-PAGE assay with Coomassie staining (**Figure S1A**), an increasing intensity was observed in the Pmp17G-linked DTSSP compared to Pmp17G alone (**Figure S1B**), and higher intensity of Pmp17G-related EGFR was found in the Pmp17-linked group compared to the Pmp17G alone group (**Figure S1C**). To confirm that Pmp17G directly interacted with EGFR in host cells, Co-immunoprecipitation (Co-IP) assays were carried out with anti-His mAbs or anti-EGFR mAbs, and Pmp17 binding to EGFR was confirmed by Western blots (**Figures 3A, B** **)**. Then, overexpression of Pmp17G and EGFR was used to validate the interaction. Finally, positive band of Pmp17G-recognized EGFR was identified with anti-HA antibody (**Figure 3C**) and anti-flag antibody (**Figure 3D**), indicating that EGFR is a receptor of Pmp17G.

### EGFR Was a Receptor During *C. psittaci* Infection

**Figure 4:**
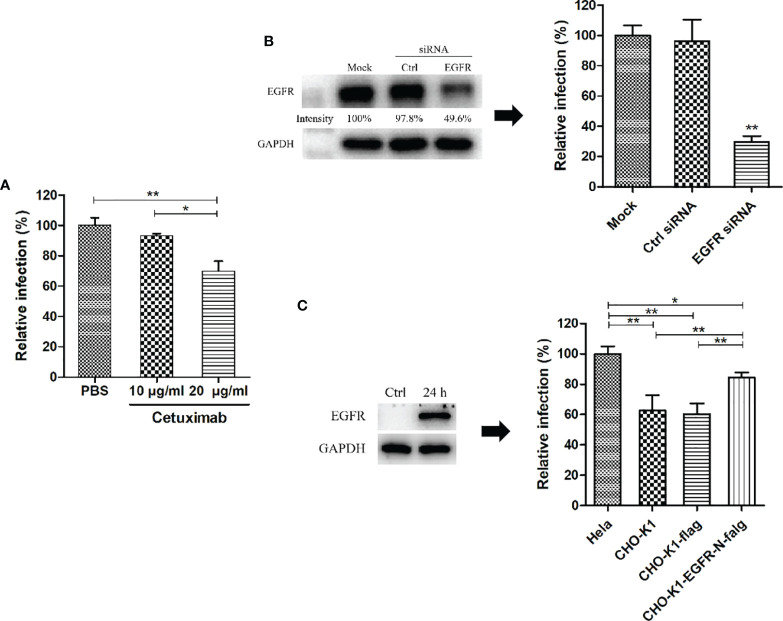
EGFR was essential for *C. psittaci* infection. **(A)** HeLa 229 cells were treated with cetuximab (10 μg/ml and 20 μg/ml) for 2 h and then inoculated with *C. psittaci* 6BC (MOI=5). Relative infection was calculated on the basis of inclusion bodies, and Relative infection (%)=(IFUs of the treated group/IFUs of the control group) ×100. **(B)** HeLa cells were transfected with EGFR siRNA (siEGFR) or control siRNA (Ctrl). Twenty-four hours after the 2nd treatment with siRNA, the cell cultures were exposed to *C psittaci* 6BC (MOI=5) and, concurrently lysed to determine efficiency of siRNA by Western blots. The intensity of the bands was quantified using ImageJ. GAPDH was used as the loading group. Intensity was determined as follows: Intensity(%)=(EGFR/GAPDH) ×100. In addition, inclusion bodies were counted and relative infection was determined as follows: Relative infection (%)=(IFUs of the siRNA-treated group/IFUs of the mock group) ×100. **(C)** CHO-K1 cells were transfected with exotic EGFR-flag, and ectopic expression of EGFR was analysed by Western blots. Twenty-four hours post-transfection, cell cultures were infected with *C. psittaci* 6BC (MOI=5). At 36-48 hpi, inclusions were measured by IIF. Relative infection was calculated using the formula: Relative infection(%)= (IFUs of the CHO-K1 group/IFUs of the HeLa 229 group) × 100. Statistical analysis was performed by one-way ANOVA, and the data were expressed as the means ± SD. Asterisks indicate statistical significance: *p < 0.05, and **p < 0.01.

To further confirm the roles of EGFR during *C. psittaci* infection, serial doses of cetuximab were used to block EGFR in host cells. After inoculation with *C. psittaci* 6BC, 20 μg/ml cetuximab was optimized for blocking *Chlamydia* infection, and a dose-dependent effect was found in a neutralization test (**Figure 4A**). After treatment with EGFR siRNA, the relative infection was reduced dramatically compared to that of the control siRNA and the mock groups (**Figure 4B**). Moreover, the relative infection was increased significantly in the EGFR overexpression group compared to that in the CHO-K1 cell lines or CHO-K1 cells transfected with the control vector (p<0.01) (**Figure 4C**), indicating that EGFR plays a major role in early *C. psittaci* infection.

### Pmp17G-Mediated Adhesion and Invasion During *C. psittaci* Infection

**Figure 5:**
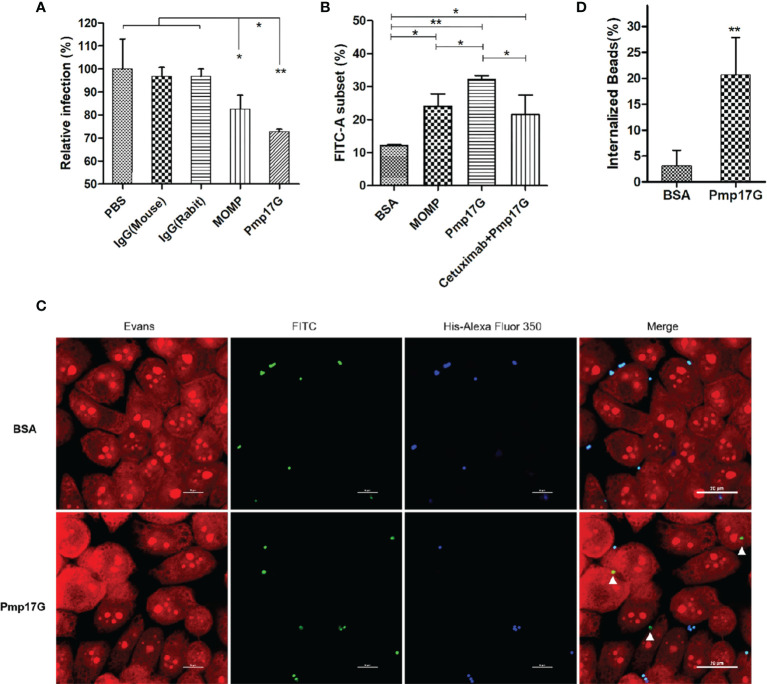
Pmp17G contributed to adhesion and invasion during *C. psittaci* infection. **(A)** *C. psittaci* 6BC was treated with anti-Pmp17G antibody or anti-MOMP antibody while IgG(mouse), IgG(rabbit) and PBS were control groups. Then, HeLa 229 cells were infected with these preparations for 36-48 h Relative infection (%)=(IFUs of the antibody-treated group/IFUs of control group) ×100. **(B)** Pmp17G adhesive capability to HeLa 229 cells. HeLa 229 cells were incubated with BSA-, MOMP- or Pmp17G-coated green fluorescent beads for 2 h at 37°C. Then, cell cultures were treated with trypsin (non-EDTA) for 3 min and washed 3 times with PBS, and the cells were resuspended in PBS. Adhesion of FITC-A subsets was measured using flow cytometry and proportion of FITC-A subsets was determined by dividing the number of FITC-labelled cells by the total number of cells. Three replicates of each measurement were performed. **(C)** Pmp17G invasion capability into HeLa 229 cells. HeLa 229 cells were incubated with BSA-, or Pmp17G-coated green fluorescent beads for 6 h at 37°C. Subsequently, the cell cultures were washed 5 times with PBS and fixed with 0.4% paraformaldehyde. Later, the extracellular binding beads were stained with 6×His-tag antibody (blue). Internalized beads displayed green. Cell structures were stained with Evans blue (red). **(D)** Internalized bead(%)=(Numbers of internalized beads/all the binding beads) ×100. Statistical analysis was performed by one-way ANOVA, and the data were expressed as the means ± SD. Asterisks indicate statistical significance: *p < 0.05, and **p < 0.01.

After *C. psittaci* 6BC was pretreated with anti-Pmp17G antibody or anti-MOMP antibody, a significant difference was found among the IgG (mouse) group, IgG(rabbit) group, the MOMP antibody-treated group and the Pmp17G antibody-treated group, suggesting that the Pmp17G antibody reduced dramatically the extent of the *Chlamydia* infection (**Figure 5A**). To monitor Pmp17G-mediated adhesive capability, an increase in the FITC-A subset was observed in the Pmp17G-coated group compared to the positive MOMP-coated group (p<0.05) or the negative BSA-coated group (p<0.01). To elucidate the role of EGFR, HeLa 229 cells were pretreated with cetuximab and then incubated with Pmp17G-coated green fluorescent beads. Bead adhesion to host cells was inhibited significantly in the cetuximab/Pmp17G group compared to the Pmp17G group (p<0.05) (**Figure 5B**). More interestingly, only Pmp17G-coated beads exhibited intracellular localization and 20.7% internalized beads were determined while BSA-labelled beads presented solely on cellular surfaces (**Figures 5C, D** **)**, suggesting that Pmp17G is not only an adhesin but also an invasin during *C.psittaci* infection.

### Pmp17G Activated EGFR Phosphorylation and Grb2 Recruitment

**Figure 6:**
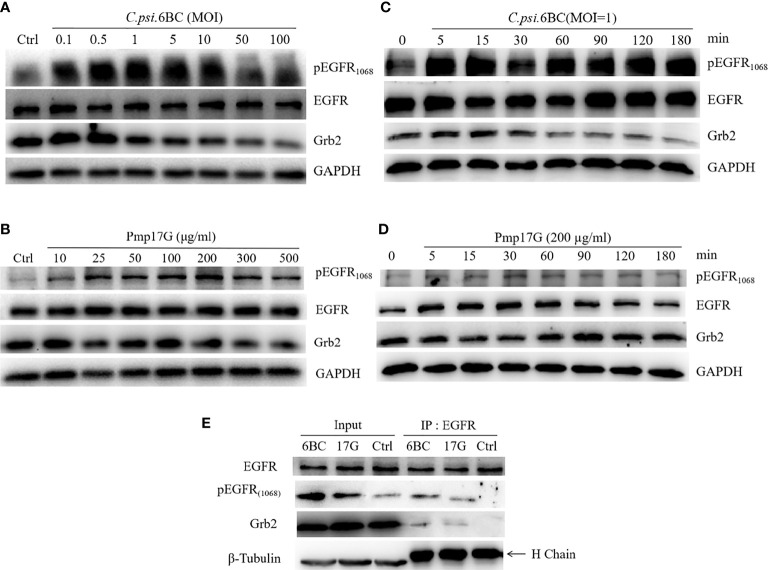
Pmp17G stimulated the phosphorylation of EGFR and downregulated Grb2 expression. **(A)** HeLa 229 cells were infected with serial doses of *C. psittaci* (MOI from 0.1 to 100), and the expression of EGFR, pY1068-EGFR and Grb2 was identified using Western blots. **(B)** HeLa 229 cells were treated with serial doses of Pmp17G (from 10 μg/ml to 500 μg/ml), and the expression of these proteins was determined using Western blots. **(C)** HeLa 229 cells were infected with *C. psittaci* (MOI=1.0), and the expression of the aforementioned proteins was identified at different time points (at 5, 15, 30, 60, 90, 120, and 180 min) by Western blots. **(D)** HeLa 229 cells were treated with 200 μg/ml of Pmp17G, and the expression levels of these proteins were determined at different time points. GAPDH was used as the loading control. **(E)** Grb2 recognized pY1068-EGFR by Co-IP assay. HeLa cells were treated with *C. psittaci* 6BC (MOI=1.0) or 200 μg/ml of Pmp17G, and IP was performed with anti-EGFR mAbs for 0.5 h at 4°C. Then, IP compounds were incubated with anti-EGFR, anti-pY1068-EGFR or anti-Grb2 antibodies, respectively and identified by Western blot assay. β-Tubulin was used as the loading control.

After HeLa 229 cells were inoculated with different doses of *C. psittaci* 6BC, phosphorylation of EGFR at site 1068 was activated in a dose-dependent manner with *C. psittaci* inoculation ranging from 0.1 to 1.0 MOI, while a gradual reduction in EGFR phosphorylation was observed with *C. psittaci* 6BC inoculation ranged from 5.0 to 100.0 MOI. Compared to pY1068-EGFR, no difference of EGFR expression was found after infection with different doses of *C. psittaci*. In contrast, Grb2 expression was downregulated in a dose-dependent manner (**Figure 6A**). Upon Pmp17G treatment, the expression of EGFR, pY1068-EGFR and Grb2 displayed similar trends (**Figure 6B**). The time-dependent effect was assessed, and the results showed that increased EGFR phosphorylation was observed with prolonged time, while a negative correlation was found between time points and Grb2 expression after exposure to EBs (**Figure 6C**). Upon Pmp17G treatment, EGFR phosphorylation increased gradually with prolonged time. However, the expression of EGFR and Grb2 displayed no difference after Pmp17G treatment (**Figure 6D** **)**. To determine whether Grb2 was translocated from the cellular reticulum to EGFR phosphorylation at site 1068 in the plasma membrane, we carried out Co-IP to determine whether EGFR binds to Grb2. Subsequently, positive bands were observed in both the *C. psittaci* group and the Pmp17G treated group (**Figure 6E**), indicating that EGFR phosphorylation might recruit Grb2 and trigger *C. psittaci* internalization.

## Discussion

Compared to *C. trachomatis*, *C. pneumoniae* and *Chlamydia muridarum* (*C. muridarum*), the pathogen *C. psittaci* infects diverse hosts, causing mild symptoms to severe pathogenicity and even mortality. Attachment and invasion to host cells are two main steps of *Chlamydia* infection. *Chlamydia* has a powerful ability to infect host cells by attaching or binding receptors. Pmps are enriched in the outer membrane protein complex of *Chlamydia* species. However, the role of PmpG-mediated tissue tropism has been poorly elucidated. In the current study, Pmp17G was found to mediate adhesive properties as an adhesin in a dependent manner by binding EGFR. More importantly, Pmp17G activated intracellular invasion in an EGFR-dependent manner during *C. psittaci* infection. Therefore, Pmp17G acts as both an adhesin and invasin during *C. psittaci* infection.

As an adhesin of *C. psittaci*, Pmp17G is able to bind to multiple host cells and promotes chlamydial adhesion in early infection. The typical time-dependent pattern was observed in HeLa 229 cells and Vero cells, with identifiable adhesion at 120 min after treatment with Pmp17G. More importantly, expression of Pmp17G enhanced *C.trachomatis* infection, implying that Pmp17G might contribute to infection of the multiple hosts and tissue tropism of *C. psittaci*. Our data corroborate a recent report that Pmp17G plays a role as an adhesin in early Chlamydial infection (15–17). However, our data are inconsistent with reports of high adhesive capacity of avian cells (DF-1 cells) and low adhesion to mammalian cells (McCoy cells) (16). However, a blocking assay indicated that there was no significant difference in relative infection among HeLa 229 cells, Vero cells and DF-1 cells, with infection in all these cells reduced from 23.03% to 29.96%. Pmps-mediated adhesion is associated with conserved 4-peptide motifs (14, 19) and oligomers (26, 27). The numbers of motifs are crucial for the adhesive capability of Pmps to certain cells, but no correlation was found between motif-mediated adhesion and inhibited infectivity in our study. Although only 3 motifs are present in recombinant Pmp17G, high adherence to HeLa 229 cells was observed in the study.

Activation of EGFR is required for the attachment and development of *Chlamydia* (25, 28, 29). Pmp17G binding to EGFR was identified by pull-down assay and mass spectrometry. The interaction of Pmp17G and EGFR was also determined by Co-IP. Adhesion was inhibited by cetuximab, an EGFR-specific antibody. Moreover, *C. psittaci* infection was reduced significantly either using cetuximab or EGFR knockdown in HeLa 229 cells. In contrast, *Chlamydia* infection was recovered by the overexpression of EGFR in CHO-K1 cell lines. Therefore, Pmp17G binding to EGFR is the first step for intracellular adhesion. The role of EGFR in *C. psittaci* infection revealed in our study is consistent with that described in previous reports of *C. pneumoniae* attachment *via* Pmp21-recognized EGFR (25) and *C. trachomatis* infection (28, 29). As an adhesion component, Pmp21 was the first identified Pmps component that interacted with EGFR during *C. pneumoniae* infection (25). In our study, the diversity of *C. psittaci* adhesion to host cells might be associated with Pmp17G-mediated EGFR affinity due to EGFR enrichment in the placenta and kidney of human tissues (30). However, EGFR abundance is unclear among HeLa 229 cells, Vero cells and DF-1 cell.The further investigation will be elucidated diverse affinity to host cells.

Additionally, Pmp17G recognition of EGFR facilitates bacterial invasion. Both Pmp17G and *C. psittaci* EBs are able to induce phosphorylation at the 1068 site of EGFR, which is consistent with previous reports of *C. pneumoniae* EBs/Pmp21 (14). Regarding EGFR phosphorylation, Pmp17G induces weaker activation than whole *C. psittaci* EBs. Once EGFR is activated, Grb2 is rapidly recruited to the plasma membrane, where it binds to phosphorylated EGFR and triggers bacterial invasion by forming the EGFR-Grb2 complex, in which Grb2 plays a role in the macropinocytic internalization pathway of EGFR in activated cells (31). Our data are consistent with a previous report that Pmp21 binding to EGFR resulted in the recruitment of both Grb2 and c-Cbl, as well as activation of downstream ERK1/2 signalling (25). In another previous report, the EGFR and TGF-β signalling pathways were found to cooperate to optimize inclusion development, cytoskeletal remodelling, and induction of the pathogenic epithelial-mesenchymal transition (EMT) during *C. trachomatis* infection (28). Functional analysis of phosphoproteome and transcriptome data confirmed the involvement of these pathways in the EMT during infection, a phenotype that was confirmed in infected cells, along with the essential roles of ERK1/2, ETS1, and ERF activation in *C. trachomatis* replication (32). However, effect of Pmp17G on inclusion development, cytoskeletal remodelling and EMT stabilization by EGFR phosphorylation remains elusive, and further work is required to discover the potential mechanism.

Taken together, our results show that *C. psittaci*-specific Pmp17G possesses adhesive properties, enabling it to serve as an adhesin to host cells. It also activates chlamydial invasion in EGFR-dependent manner, activating EGFR through Tyr1068 phosphorylation and forming the EGFR-Grb2 complex, contributing to intracellular attachment and internalization during *C. psittaci* infection. Pmp17G is a critical biomarker due to its roles as an adhesin and invasion. However, further investigation is urgently needed to identify the target mechanism for combatting *Chlamydia* infection.

## Data Availability Statement

The original contributions presented in the study are included in the article/**Supplementary Material**. Further inquiries can be directed to the corresponding author.

## Author Contributions

Conceptualization: CH and JH. Methodology: XL and ZZ. Investigation: XL, ZZ, and YW. Visualization: XL and ZZ. Supervision: CH and JHH Writing-original draft: XL, ZZ. Writing—review and editing: CH. All authors contributed to the article and approved the submitted version.

## Funding

This study was funded by the National Natural Science Foundation of China (grant 31672517) and Taishan Scholar Project of Shandong province (grant ts201511084).

## Conflict of Interest

The authors declare that the research was conducted in the absence of any commercial or financial relationships that could be construed as a potential conflict of interest.

## Publisher’s Note

All claims expressed in this article are solely those of the authors and do not necessarily represent those of their affiliated organizations, or those of the publisher, the editors and the reviewers. Any product that may be evaluated in this article, or claim that may be made by its manufacturer, is not guaranteed or endorsed by the publisher.
