# Supplementary material for: Polymorphic Membrane Protein 17G of Chlamydia psittaci Mediated the Binding and Invasion of Bacteria to Host Cells by Interacting and Activating EGFR of the Host
Source: Front Immunol. 2022 Jan 31;12:818487. doi: 10.3389/fimmu.2021.818487 (PMC8841347; doi:10.3389/fimmu.2021.818487)
Supplement: Supplementary file 1 [file DataSheet_1.docx]

Supplementary Material

# Supplementary Figures


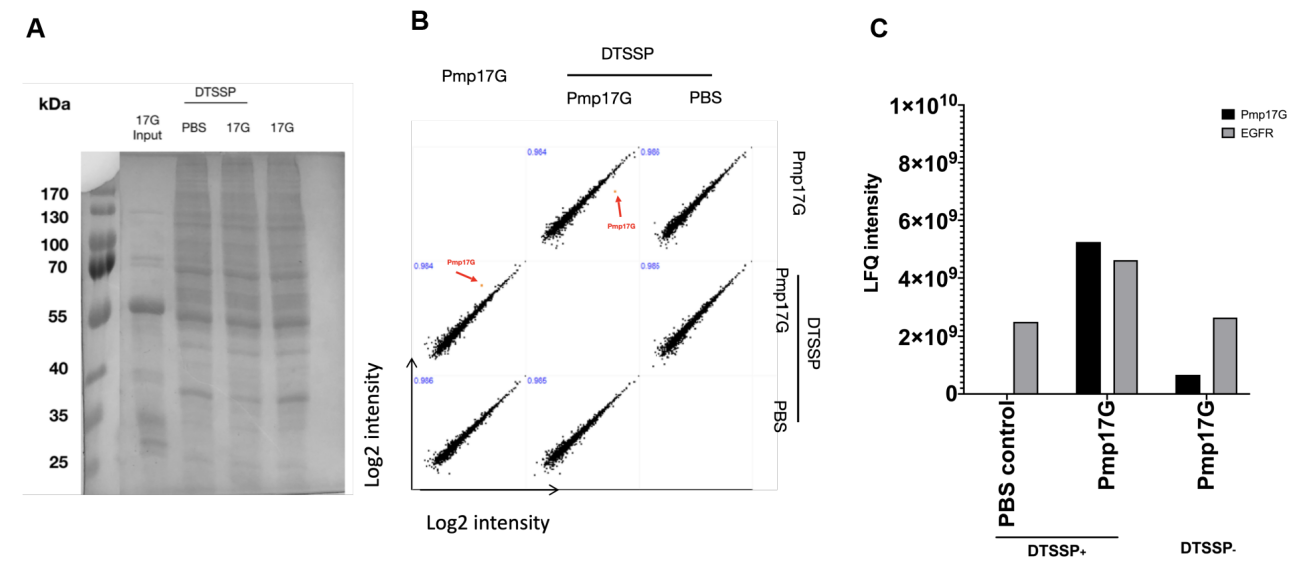


**Supplementary Figure 1.** Screening Pmp17G-binding proteins in HeLa 229 cells. (A) HeLa 229 cells were incubated with Pmp17G and then subjected to a Ni-NTA pull-down assay. No significant targeted proteins were identified using DTSSP. (B) Intensity analysis was found with Pmp17G alone and Pmp17G-linked DTSSP by mass spectrometry. (C) An increasing intensity of label-free quantitation (LFQ) was observed with epidermal growth receptor (EGFR) and Pmp17G-linked DTSSP compared to Pmp17G alone.


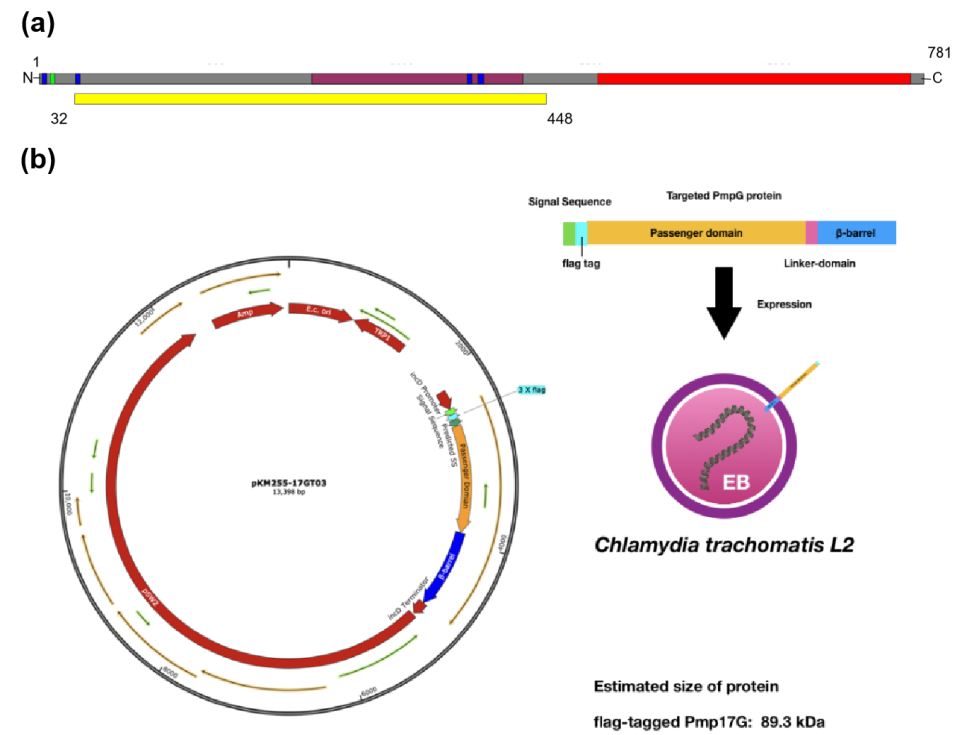


**Supplementary Figure 2.** Schematic domains of Pmp17G protein in *C. psittaci*. Pmp17G protein was analyzed using MOTIF website at https://www.genome.jp/tools/motif/ (full-length, gray mark; target domain, yellow mark: GAA (I, L, V) motifs, green mark; FxxN motifs, blue mark; central PMP-M region, purple mark; autotransporter β-domain,red mark). (b) Construction of *C. trachomatis* L2 transformation vector containing *C. psittaci pmp17G* gene

# Supplementary Table

**Supplementary Table 1.** Primers and templates used in this study.

| Cloning of pET28a-MOMP | | |
| --- | --- | --- |
| **Primers:** | MOMP-F | 5’-CATG**CCATGG**atgAAAAAACTCTTGAAATCGGC-3’ (*Sal* Ⅰ) |
|  | MOMP-R | 5’-GC**GTCGAC**TTGAATCTGAATTGAGCATTCAT-3’(*Nco* Ⅰ) |
| **Templates:** | *Chlamydia psittaci* 6BC chromosome, complete genome | |
| **GenBank:** | CP002549.1 | |
|  | | |
| Cloning of pET28a-Pmp17G | | |
| **Primers:** | Pmp17G-n-F-1 | 5’-GC**GTCGAC**atgATCTTCCAAGGCAATAAAGTCA-3’ (*Sal* Ⅰ) |
|  | Pmp17G-n-R-1 | 5’-G**GAATTC**TGCAGATACCCATTTGATTTCGAA -3’(*EcoR* Ⅰ) |
| **Templates:** | *Chlamydia psittaci* 6BC chromosome, complete genome | |
| **GenBank:** | CP002549.1 | |
|  |  | |
| Cloning of pKM255::Pmp17G | | |
| **Primers:** | 17GT0301 | 5’-CAAGGATCATGATATTGATTACAAAGACGATGACGATAA  GATGACTTTCTCCAATAACTCCTCAG-3’ |
|  | 17GT0104 | 5’-GTCTCCCTCCTCTTTTTCCTACGCGAATCACATGTCATC  CGAATTTGTACCTGCCTCCTATG-3’ |
| **Templates:** | *Chlamydia psittaci* 6BC chromosome, complete genome | |
| **GenBank:** | CP002549.1 | |
|  | | |
| Cloning of pCMV-Pmp17G-HA | | |
| **Primers:** | Pmp17G-n-F-2 | 5’-GC**GTCGAC**atgATCTTCCAAGGCAATAAAGTCA-3’ (*Sal* Ⅰ) |
|  | Pmp17G-n-R-2 | 5’-GC**TCTAGA**TGCAGATACCCATTTGATTTCGAA -3’(*Xba* Ⅰ) |
| **Templates:** | *Chlamydia psittaci* 6BC chromosome, complete genome | |
| **GenBank:** | CP002549.1 | |
|  |  | |
| Cloning of pCMV-EGFR-N-flag | | |
| **Primers:** | EGFR-ex-F | 5’-GC**GTCGAC**atgCTGGAGGAAAAGAAAGTTTG-3’(*Sal* Ⅰ) |
|  | EGFR-ex-R | 5’-GC**TCTAGA**ATGGCACAGGTGGCACACATGG-3’(*Xba* Ⅰ) |
| **Templates:** | *Homo sapiens* epidermal growth factor receptor (EGFR), transcript variant 5, mRNA | |
| **GenBank:** | NM_001346897.2 | |
